# The month of July: an early experience with pandemic influenza A (H1N1) in adults with cystic fibrosis

**DOI:** 10.1186/1471-2466-10-8

**Published:** 2010-02-25

**Authors:** Megan W France, Szeanna Tai, Phillip J Masel, Vanessa L Moore, Tracy L McMahon, Alexander J Ritchie, Scott C Bell

**Affiliations:** 1 The Prince Charles Hospital Adult Cystic Fibrosis Centre, Rode Rd, Chermside, Queensland, 4032, Australia; 2 The University of Queensland, St Lucia, Queensland, 4072, Australia

## Abstract

**Background:**

Pandemic Influenza A (H1N1) 2009 is a novel viral infection that emerged in March 2009. This is the first report addressing the clinical course of patients with cystic fibrosis (CF) and H1N1 infection.

**Methods:**

All patients with an influenza-like illness (ILI) attending our adult centre during July 2009 were identified. Baseline respiratory function, nutritional status, approach to management and short-term clinical course were recorded.

**Results:**

Most patients experienced a mild course and were able to be managed with antiviral agents as an outpatient. Robust infection control policies were implemented to limit transmission of H1N1 infection within our CF centre. Patients with severe lung disease, poor baseline nutritional reserve and presenting with more than 48 hours of ILI experienced a more severe course. Prompt antiviral therapy within the first 48 hours of illness may have been important in improving outcomes.

**Conclusions:**

This observational study demonstrates that most adults with CF with H1N1 infection had mild clinical courses and recovered rapidly.

## Background

H1N1 is a novel influenza virus containing a combination of gene segments from North American and Eurasian swine lineages [1]. The first reported cases of human H1N1 infection emerged from Mexico in March 2009 [2]. Widespread availability of airline travel contributed to the novel virus' ability to move quickly to other continents [3].

Viral infections are well recognised precipitants of acute deterioration in the clinical status of patients with cystic fibrosis [4-9]. Increased respiratory symptoms and hospitalisation and reduced pulmonary function are seen during a viral infection [8]. There have been some reports that a longer-term deleterious effect may also be attributable to severe viral infection [5,8].

Viral infections also contribute to clinical deterioration by altering lower airway defences against bacterial infection. For example, new Pseudomonas aeruginosa infection has been noted to peak during winter periods when viral infections are common [8,10]. RSV have been shown to compromise airways defences via damage to airway epithelium and depletion of the periciliary layer (PCL) [11]. Influenza A virus has also been shown to accelerate neutrophil apoptosis [12].

This is the first report of the outcomes seen in CF patients infected with Pandemic (H1N1) 2009. Our experience provides some insights in the management of H1N1-infected patients with CF.

## Method

Patients with an influenza-like illness (ILI) at our centre in July 2009 were identified. An ILI was defined as recent fever, with one or more of the following: sore throat, myalgias, rhinorrhea, headache and gastrointestinal symptoms. CF centre nursing staff performed the swab if a clinical diagnosis of ILI had been made.

Three dry sterile swabs were used to collect samples; two from the nasopharynx and one from the posterior oropharynx. Molecular detection using a multiplex assay for influenza A was performed and when positive, a H1N1-specific polymerase chain reaction (PCR) assay was then performed by the Molecular Viral Laboratory of QLD Pathology [13].

After 22 July 2009, patients were treated on clinical suspicion and no longer swabbed, in accordance with the policy of our state government health authority. Subsequently, only people presenting with ILI from the following groups were tested: pregnant women, indigenous people, hospitalised patients and healthcare workers.

This study was approved by The Prince Charles Hospital Research, Ethics and Governance Committee. Individual patient consent for the study was not required by the Ethics Committee.

## Results

Twelve of our 270 patients (4.4%) presented with an ILI during the month of July 2009, including 11 confirmed by swab and one who was treated after a clinical diagnosis of H1N1. Only one patient reported contact with a known H1N1- infected person. All infections were community-acquired and no patients reported recent travel overseas. During this period a further five patients presented with ILI and subsequently had negative H1N1 2009 PCR assays. One of these patients had a positive PCR for respiratory syncytial viral infection, and the other four patients did not have other respiratory viral infections detected.

Two patients were admitted after initial management at home with oseltamivir and antibiotic therapy, including one patient admitted on day four of symptoms with large volume haemoptysis and moderate infective exacerbation of bronchiectasis. He had co-infection with human metapneumovirus and sought early discharge from hospital. The second patient had a diagnosis of H1N1 infection made on the basis of clinical presentation and treated in the community with oseltamivir for five days. Within 36 hours of completing therapy, she was admitted with a recrudescence of viral symptoms. Swabs were collected and confirmed the presence of H1N1 2009 infection. She was recommenced on oseltamivir therapy for a further five days and was discharged 48 hours after hospitalisation.

Symptoms at presentation were variable. Fever (92%), increased cough (92%), rhinorrhea (75%) and myalgias (67%) were most commonly seen. Headache and sore throat occurred in one third of patients. There were 7 males and 5 females in the cohort with a mean FEV_1_% predicted of 66% (SD 20) and a mean BMI of 22 (SD 5) kg/m^2^. The mean age of our H1N1 affected CF patients was notably lower than our total CF clinic mean age ie. 22 years (SD 3) compared with 28 years (SD 9). Baseline characteristics of the patients and their management are summarised in Additional File 1. Notably all patients reported here had chronic *P. aeruginosa *infection. The clinical course was variable however overall was mild. Seven of the patients required admission to hospital and four of these were hospitalised for a period of 7 days or less.

Patients with ILI were commenced immediately on a five day course of oseltamivir 75 mg twice daily. In accordance with current recommendations for other high risk groups, all patients were prescribed oseltamivir even if they presented with symptoms of more than 48 hours duration. Oseltamivir was well tolerated with no treatment cessation due to side effects.

## Discussion

The outcomes for patients with H1N1 2009 infection and CF have not been previously described. Known risk groups including pregnant women, people with a chronic illness, including respiratory disease, indigenous groups, obese people and immunosuppressed hosts [14]. The first Australian case of H1N1 infection was reported on 8 May 2009 [15].

World Health Organisation data suggests that the majority of cases are occurring in people aged 12 to 17 years and the USA reports that 60% of cases are 18 years or less [16]. The median age of patients with H1N1 infection in Australia is 21 years [17]. Our series supports this observation with the mean age of our H1N1 affected group lower than that of our CF clinic.

Symptoms in the 12 patients reported in our series were variable. The diagnosis on clinical grounds alone is more challenging in patients with a chronic respiratory symptomatology. One patient developed more severe ILI symptoms after the cessation of oseltamivir, which may represented two discrete viral infections or may have had a recrudescence of H1N1 infection at the cessation of antiviral treatment. Patients with severe airflow obstruction experienced more severe respiratory exacerbations, especially if they presented with ILI of more than forty-eight hours duration.

The experience of our centre provides an opportunity to formulate some suggestions for management CF patients during an influenza pandemic. Firstly, ensuring that CF centre staff are skilled in performing nasopharyngeal swabs allows rapid testing and limit movement of the patient throughout the hospital. Secondly, encouraging patients with ILI to notify the CF centre prior to presenting to the outpatient clinic allows rapid isolation of these patients. Early written correspondence with the patient group will assist with education of patients regarding best practice with ILI. Thirdly, establishment of practical policies for management of suspected H1N1 infected patients allows all staff access to rapid decision-making regarding management, 24 hours a day including strict isolation with droplet precautions. Our institution supported by the State Government, developed protocols for management of all suspected H1N1 patients. These were readily able to be adapted to our CF population. A policy for the early use of antiviral agents may offer the greatest opportunity for avoidance of severe H1N1-related disease.

By the end of July 21,668 cases of H1N1 2009 had been reported across Australia [18] and by November this had increased to greater than 37,000 cases [19]. Deaths attributable to H1N1 2009 infection in Australia were 189 by November 2009 [19]. This is likely to be an underestimate of the true numbers of cases [20]. The frequency of ILI seen in our patient group was lower than may have been expected, given the large number of cases in the community during July 2009. There may be patients with ILI who did not notify the CF centre and self-managed their illness. Patients may also have had H1N1 infection which was undetected as they have attributed the symptoms to their underlying respiratory illness and not sought medical review. Another possible explanation may be that CF patients are more attentive to hand hygiene and avoidance of people with viral symptoms than may be expected of the general community.

As a result of the vast number of nasopharyngeal swab tests performed during July 2009 a change in H1N1 testing was required and subsequently only patients with ILI and risk factors for an adverse outcome (e.g. pre-existing cardiorespiratory disease) had viral testing performed [20]. Patients without ILI were not tested for H1N1 2009 during the pandemic period. Consequently, the rates of asymptomatic or minimally symptomatic H1N1 infection in this population could not be estimated. Furthermore, only one patient was detected to have another respiratory virus (human metapneumovirus) as generally when H1N1 testing was positive additional respiratory viral testing was not performed. During the month of August, another 13 cases of H1N1 infection were seen with similar clinical impact in the initial cohort described here.

Since this analysis was performed, the H1N1 vaccination has been released (6^th ^October 2009) and has been made freely available by the Commonwealth Government to all Australians. The Adult Cystic Fibrosis Centre at The Prince Charles Hospital has notified patients attending the Centre of its availability during hospital attendance, and we have vaccinated all consenting patients who did not have H1N1 2009 infection and this process is ongoing. Despite the impact of the recent pandemic, some patients have elected not to be vaccinated to date.

## Conclusions

Despite significant symptoms, many of our patients were successfully managed in the community with prompt commencement of oseltamivir and supported by appropriate antibiotic therapy. Patients with severe lung disease, poor nutritional reserve and presenting late with ILI experienced severe exacerbations characterised by hypoxaemia and required prolonged hospitalisation.

## Competing interests

The authors declare that they have no competing interests.

## Authors' contributions

MWF - participated in study design, compiled clinical data, and drafted the manuscript. SAT - compiled clinical data. PJM - compiled clinical data. VLM - compiled clinical data. TM - compiled clinical data. AJR - compiled clinical data. SCB - conceived the study, participated in the study design and coordination. All authors read and approved the final manuscript.

## Pre-publication history

The pre-publication history for this paper can be accessed here:

http://www.biomedcentral.com/1471-2466/10/8/prepub

## Supplementary Material

Additional file 1 **Table 1**. Baseline characteristics and early outcomes of patients with influenza-like illness. Click here for file

## References

[B1] Garten RJ Davis CT Russell CA Shu B Lindstrom S Balish A Antigenic and genetic characteristics of swine-origin 2009 A(H1N1) influenza viruses circulating in humans Science (New York, NY) 2009 325 5937 197 201 10.1126/science.1176225 PMC3250984 19465683

[B2] Chowell G Bertozzi SM Colchero MA Lopez-Gatell H Alpuche-Aranda C Hernandez M Severe Respiratory Disease Concurrent with the Circulation of H1N1 Influenza N Engl J Med 2009 361 7 674 9 10.1056/NEJMoa0904023 19564633

[B3] Khan K Arino J Hu W Raposo P Sears J Calderon F Spread of a novel influenza A (H1N1) virus via global airline transportation N Engl J Med 2009 361 2 212 4 10.1056/NEJMc0904559 19564630

[B4] Armstrong D Grimwood K Carlin JB Carzino R Hull J Olinsky A Severe viral respiratory infections in infants with cystic fibrosis Pediatr Pulmonol 1998 26 6 371 9 10.1002/(SICI)1099-0496(199812)26:6<371::AID-PPUL1>3.0.CO;2-N 9888211

[B5] Collinson J Nicholson KG Cancio E Ashman J Ireland DC Hammersley V Effects of upper respiratory tract infections in patients with cystic fibrosis Thorax 1996 51 11 1115 22 10.1136/thx.51.11.1115 8958895 PMC1090523

[B6] Conway SP Simmonds EJ Littlewood JM Acute severe deterioration in cystic fibrosis associated with influenza A virus infection Thorax 1992 47 2 112 4 10.1136/thx.47.2.112 1549818 PMC463587

[B7] Ong EL Ellis ME Webb AK Neal KR Dodd M Caul EO Infective respiratory exacerbations in young adults with cystic fibrosis: role of viruses and atypical microorganisms Thorax 1989 44 9 739 42 10.1136/thx.44.9.739 2588211 PMC462055

[B8] van Ewijk BE Zalm MM van der Wolfs TF Ent CK van der Viral respiratory infections in cystic fibrosis J Cyst Fibros 2005 2 31 6 10.1016/j.jcf.2005.05.011 PMC7105219 15964785

[B9] Wat D Gelder C Hibbitts S Cafferty F Bowler I Pierrepoint M The role of respiratory viruses in cystic fibrosis J Cyst Fibros 2008 7 4 320 8 10.1016/j.jcf.2007.12.002 18255355 PMC7105190

[B10] Johansen HK Hoiby N Seasonal onset of initial colonisation and chronic infection with Pseudomonas aeruginosa in patients with cystic fibrosis in Denmark Thorax 1992 47 2 109 11 10.1136/thx.47.2.109 1549817 PMC463585

[B11] Tarran R Button B Picher M Paradiso AM Ribeiro CM Lazarowski ER Normal and cystic fibrosis airway surface liquid homeostasis. The effects of phasic shear stress and viral infections J Biol Chem 2005 280 42 35751 9 10.1074/jbc.M505832200 16087672 PMC2924153

[B12] Colamussi ML White MR Crouch E Hartshorn KL Influenza A virus accelerates neutrophil apoptosis and markedly potentiates apoptotic effects of bacteria Blood 1999 93 7 2395 403 10090951

[B13] Whiley DM Bialasiewicz S Bletchly C Faux CE Harrower B Gould AR Detection of novel influenza A(H1N1) virus by real-time RT-PCR J Clin Virol 2009 45 3 203 4 10.1016/j.jcv.2009.05.032 19515611

[B14] Intensive Care Patients with Severe Novel Inlfuenza A (H1N1) Virus Infection MMWR 2009 58 27 749 52 19609249

[B15] Australian Government Situation Report- Pandemic (H1N1) 2009 http://www.healthemergency.gov.au/internet/healthemergency/publishing.nsf/Content/bulletins-4-10-may09 cited 2010 4th February

[B16] Mossad SB The resurgence of swine-origin influenza A (H1N1) Cleve Clin J Med 2009 76 6 337 43 10.3949/ccjm.76a.09047 19487554

[B17] Kelly H Grant K Interim analysis of pandemic influenza (H1N1) 2009 in Australia: surveillance trends, age of infection and effectiveness of seasonal vaccination Euro Surveill 2009 14 31 10.2807/ese.14.31.19288-en 19660248

[B18] Australian Government Situation Report- Pandemic (H1N1) 2009 http://www.healthemergency.gov.au/internet/healthemergency/publishing.nsf/Content/bulletins-27-31-july09 cited 2010 4th February

[B19] Australian Government Situation Report- Pandemic (H1N1) 2009 http://www.healthemergency.gov.au/internet/healthemergency/publishing.nsf/Content/bulletins-26Oct-1Nov cited 2010 4th February

[B20] Kotsimbos T Waterer G Jenkins C Kelly PM Cheng A Hancox RJ Influenza A/H1N1_09: Australia and New Zealand's Winter of Discontent American journal of respiratory and critical care medicine 2010 181 4 300 6 10.1164/rccm.200912-1878CP 20130145

