# Supplementary material for: The month of July: an early experience with pandemic influenza A (H1N1) in adults with cystic fibrosis
Source: BMC Pulm Med. 2010 Feb 25;10:8. doi: 10.1186/1471-2466-10-8 (PMC2837636; doi:10.1186/1471-2466-10-8)
Supplement: Additional file 1 — Table 1. Baseline characteristics and early outcomes of patients with influenza-like illness. [file 1471-2466-10-8-S1.DOC]

**Table 1 - Baseline characteristics and early outcomes of patients with** influenza-like illness.

| **Patient** | **Gender** | **Age** | **Number of symptomatic days prior to presentation** | **Date of positive test** | **Baseline FEV_1_% predicted*** | **Baseline BMI*** | **Days of iv antibiotic therapy^#^** | **Usual bacterial pathogen** | **IP/OP therapy** | **Days admitted** | **Change in FEV_1_ % post-H1N1 infection (as at 28 August 2009)** |
| --- | --- | --- | --- | --- | --- | --- | --- | --- | --- | --- | --- |
| 1 | Male | 24 | 1 | 01/07/09 | 81 | 23.8 | 0 | Psa | IP | 7 | 2% |
| 2 | Female | 17 | 3 | 02/07/09 | 68 | 22.1 | 10 | Psa | IP | 3 | +11% |
| 3 | Female | 22 | 2 | 04/07/09 | 70 | 20.1 | 42 | Psa | OP | N/A | -7% |
| 4 | Male | 24 | 1 | 09/07/09 | 35 | 20.9 | 54 | Psa, Bcc | OP | N/A | -1% |
| 5 | Male | 21 | 2 | 13/07/09 | 75 | 27.8 | 3 | Psa | IP | 7 | N/A |
| 6 | Male | 25 | 1 | 15/07/09 | 95 | 23.2 | 0 | Psa | OP | N/A | 0% |
| 7 | Male | 20 | 1 | 15/07/09 | 75 | 17.7 | 27 | Psa | OP | N/A | -8% |
| 8 | Female | 21 | 5 | 20/07/09 | 36 | 17.5 | 20 | Psa | IP | 14 | 0% |
| 9 | Male | 26 | 3 | 20/07/09 | 35 | 16.3 | 83 | Psa | IP | 24 | -7% |
| 10 | Female | 19 | 4 | 31/07/09**^¥^** | 83 | 32.5 | 0 | Psa | OP; IP§ | 3 | -7% |
| 11 | Female | 18 | 5 | N/T | 69 | 23.6 | 55 | Psa | OP | N/A | -3% |
| 12 | Male | 28 | 1 | 29/07/09**^§^** | 69 | 23.2 | 14 | Psa | OP; IP§ | 11 | -17% |

**Abbreviations**: N/T- Not tested; iv- Intravenous; FEV1- forced expiratory volume over 1 second; BMI- body mass index; Psa- *Pseudomonas aeruginosa*; Bcc- *Burkholderia cepacia complex* which in this case was *B. multivorans;* N/A- not available at time of reporting.

*Indicates best result from the past 12 months. Not performed at review with ILI because of infection control protocols.

# Cumulative data for 12 months preceding H1N1 infection.

§ Tested by private laboratory. Co-infected with Human Metapneumovirus.

¥ Treated on clinical grounds initially; retested when unwell again after cessation of oseltamivir.
